# Genomic analysis of *Neisseria meningitidis* carriage isolates during an outbreak of serogroup C clonal complex 11, Tuscany, Italy

**DOI:** 10.1371/journal.pone.0217500

**Published:** 2019-05-28

**Authors:** Luigina Ambrosio, Arianna Neri, Cecilia Fazio, Gian Maria Rossolini, Paola Vacca, Eleonora Riccobono, Fabio Voller, Alessandro Miglietta, Paola Stefanelli

**Affiliations:** 1 Department of Infectious Diseases, Istituto Superiore di Sanità, Rome, Italy; 2 Department of Experimental and Clinical Medicine, University of Florence, Florence, Italy; 3 Clinical Microbiology and Virology Unit, Careggi University Hospital, Florence, Italy; 4 Regional Health Agency of Tuscany, Epidemiologic Observatory, Florence, Italy; 5 Units of Epidemiology and Preventive Medicine, Central Tuscany Health Authority, Florence, Italy; Universidad Nacional de la Plata, ARGENTINA

## Abstract

**Background:**

In 2015–2016, a cross-sectional carriage survey was performed in Tuscany Region, Italy, during an outbreak of invasive meningococcal disease due to *Neisseria meningitidis* serogroup C clonal complex 11 (MenC:cc11). This study aims to evaluate the genomic profile of meningococcal carriage isolates collected during the survey.

**Methods:**

Whole-genome sequencing (WGS) was performed using Illumina MiSeq on 85 cultivated meningococcal carriage isolates received at the Dept. of Infectious Disease, National Institute of Health (Istituto Superiore di Sanità, ISS), as National Reference Laboratory (NRL) for Invasive Meningococcal Disease (IMD). *De novo* assembled genomes were scanned by the BIGSdb platform to assign: the genotypic profiles, the cgMLST, the vaccine antigen variants and allele types of antimicrobial resistance associated genes, together with denitrification pathway loci.

**Results:**

Capsule null and non-groupable meningococci accounted for 52.9% and 10.6%, respectively. Among the remaining carriage isolates, serogroup B was the predominant (71.0%). Serogroup C meningococci were culture negative and unavailable for WGS. Overall, 64 genotypic profiles were identified and, based on cgMLST, isolates clustered according to clonal complexes. Eight isolates (9.4%) harbored at least one gene encoding a 4CMenB vaccine antigen. Mutated *penA* alleles were found in more than 82%. Finally, complete *aniA* and *norB* coding sequences were detected among 71.8% of carriage isolates.

**Conclusions:**

Meningococcal carriage isolates collected during the MenC:cc11 outbreak were characterized by an extensive genetic diversity. The lack of outbreak-related isolates among carriage might be attributable to the high transmissibility with low duration of colonization of MenC:cc11 meningococci.

## Introduction

*Neisseria meningitidis* (also known as meningococcus) can be considered a common commensal bacterium of the human pharynx, which represents its natural reservoir [1]. Pharyngeal carriage prevalence is age related, increasing through adolescence [2]. Occasionally, meningococci can invade the bloodstream and other normally sterile sites, leading to invasive meningococcal disease (IMD), whose most frequent clinical presentations are sepsis and meningitis.

In Europe, infants and young children are the most affected, followed by adolescents and young adults [3]. Only a minority of strains, referred to as hyper-invasive, are responsible for the majority of IMD cases worldwide [4]. It is reasonable to assume that such hyper-invasive strains are transmitted through respiratory droplets from asymptomatic carriers more frequently than from patients with IMD [1]. However, the relationship between carriage and development of IMD is not fully understood.

*N*. *meningitidis* serogroup C clonal complex 11 (MenC:cc11), strain C:P1.5–1,10–8:F3-6:ST-11 (cc11), has been reported to cause IMD outbreaks among men who have sex with men (MSM), i.e., in Germany and in France [5,6]. Moreover, those meningococcal C strains belonging to cc11 are often associated with urogenital infections [7–9]. The survival in this anatomic district seems to be associated with the ability to grow under anaerobic conditions thanks to the denitrification pathway, consisting of genes coding for a nitrite reductase (*aniA*) and a nitric oxide reductase (*norB*) [10].

Although IMD incidence is historically low in Italy, with an annual average of 0.3 cases per 100,000 between 2015 and 2017 [11], small C:P1.5–1,10–8:F3-6:ST-11 (cc11) outbreaks occurred in the country since 2008 [12, 13]. Most of the strains belonging to the hyper-invasive cc11 currently circulating in Italy showed a reduced susceptibility to penicillin G [14].

Between 2015–2016, Tuscany Region reported an unexpected increase of IMD cases due to the meningococcal C:P1.5–1,10–8:F3-6:ST-11 (cc11) strain [15–17]. As already described [18], several initiatives and studies were implemented in the Region, of which: 1) a reactive vaccination campaign with a single dose of the tetravalent ACYW conjugate vaccine or monovalent MCC vaccine, targeting the population aged between 11 and 45 years and, from December 2016, discos and lesbian, gay, bisexual, and transgender (LGBT) associations; 2) a cross-sectional carriage survey with the aim to characterize meningococcal carriage prevalence and related risk factors in the outbreak context [19].

As already published [19], 110 carriage samples resulted positives for *N*. *meningitidis* and 85 of them were cultivated in vitro. Hereby, all the 85 culture positive carriage meningococci were further characterized to evaluate the genomic profile.

## Materials and methods

### Ethics considerations

Ethical approval was obtained by the Regional Ethic Committee of Tuscany (registration no. 965) [19]. A written informed consent was signed by all the participants and by the parents/legal guardians of subjects aged less than 18 years.

### Whole-genome sequencing and typing

Eighty-five cultivated carriage isolates [19] were sent to the Italian National Reference Laboratory (NRL) for Invasive Meningococcal Diseases (IMD) at the National Institute of Health (Istituto Superiore di Sanità, ISS) for genomic investigation.

Whole-genome sequencing (WGS) was performed as previously described [15]. *De novo* assembled genomes have been uploaded onto BIGSdb platform, hosted at PubMLST.org (http://pubmlst.org/neisseria/), and analyzed through the gene-by-gene annotation approach [20]. In case of new alleles or incomplete loci, single gene sequencing and manual curation were performed. According to designation tools included in the *Neisseria* pubMLST website, isolates were characterized by capsular genogrouping, finetyping of the outer membrane proteins PorA and FetA and multilocus sequence typing (MLST). Such information defines the genotypic profile as follows: genogroup: PorA (P1). VR1,VR2: FetA (F)VR: ST (cc). Phylogenetic analysis was performed by core genome MLST (cgMLST) v1.0 [21] on 26 carriage isolates of this study plus 37 invasive meningococci, sharing the same clonal complex, isolated in 2016 in Italy (Table A in S1 Appendix). Incomplete loci were automatically removed from the distance matrix, and the results were visualised as neighbour-net networks, generated by SplitsTree4 (version 4.13.1) [22]. Genes encoding MenB vaccines antigens (fHbp, NHBA, NadA and PorA VR2) were profiled and the Bexsero antigen sequence types (BASTs) were assigned. Moreover, *penA* gene, which encodes penicillin-binding protein 2, and genes involved in denitrification pathway, *aniA* (reference number NEIS1549) and *norB* (reference number NEIS1548), were typed.

## Results

As shown in Table 1, 52.9% (45/85) carriage isolates were capsule null (*cnl*) and 10.6% (9/85) non-groupable (NG). The remaining 36.5% (31/85) displayed a complete capsule locus (*cps*): 22 were B (MenB 71.0%; 22/31), 7 Y (MenY 22.6%; 7/31), 1 E (MenE 3.2%; 1/31) and 1 Z (MenZ 3.2%; 1/31). The analysis of allelic variants within the capsule locus revealed that 9 MenB, 6 MenY and the unique MenZ harbored intact coding sequences in the *cps* locus, while the remaining isolates presented premature stop codons in one or more *cps* genes that would result in the loss of encapsulation. No meningococcal C (MenC) isolates among the samples were cultivated during the survey.

**Table 1:** Molecular characteristics of 85 meningococcal carriage isolates.

	PorA type	FetA type	Sequence Type (ST)	clonal complex (cc)	N. of isolates
***cnl* (n = 45)**	P1.18–4,25	F4-49	ST-1136	cc1136	7
	P1.18–4,25	F4-1	ST-1136	cc1136	4
	P1.18–4,25	F1-29	ST-1136	cc1136	2
	P1.18–4,25	F2-1	ST-1136	cc1136	1
	P1.18–4,25	F5-18	ST-1136	cc1136	1
	P1.18–4,25	NA^†^	ST-1136	cc1136	1
	P1.18–4,25	F4-49	ST-11738	cc1136	1
	P1.18–4,25	F5-18	ST-12470	cc1136	1
	P1.18–4,25–37	Δ*fetA*^‡^	ST-13193	cc1136	1
	P1.18,25–14	Δ*fetA*^‡^	ST-823	cc198	3
	P1.18,25–14	NA^†^	ST-823	cc198	2
	P1.18,25–14	F5-5	ST-823	cc198	1
	P1.18,25–54	Δ*fetA*^‡^	ST-823	cc198	1
	P1.18,25–32	F5-5	ST-823	cc198	1
	P1.18,25–88	Δ*fetA*^‡^	ST-823	cc198	1
	P1.18,25–88	NA^†^	ST-823	cc198	1
	P1.18,25–89	Δ*fetA*^‡^	ST-823	cc198	1
	P1.18,25–1	F5-5	ST-198	cc198	1
	P1.18–45,25–11	F5-5	ST-198	cc198	1
	P1.7,30	F1-2	ST-53	cc53	1
	P1.7,30	F1-5	ST-53	cc53	1
	P1.7,30	F1-145	ST-53	cc53	1
	P1.7,30–1	F1-2	ST-53	cc53	1
	P1.7,30–3	F1-59	ST-53	cc53	1
	P1.7–2,30	F1-2	ST-53	cc53	1
	P1.7–2,30–2	F1-5	ST-53	cc53	1
	P1.7–2,30–3	F1-2	ST-53	cc53	1
	P1.7–2,30–1	F1-7	ST-11167	cc53	2
	P1.7,30	F1-20	ST-12473	cc53	1
	P1.7–2,30–3	F1-7	ST-13194	cc53	1
	P1.18,10–2	F1-84	ST-12471	cc41/44	1
**MenB (n = 22)**	P1.21,16–36	F5-8	ST-3327	cc865	3
	P1.5–2,16–36	F5-8	ST-3327	cc865	1
	P1.18,25–14	F5-8	ST-3327	cc865	1
	P1.19,15	F1-84	ST-414	cc41/44	1
	P1.18,25	F1-84	ST-414	cc41/44	1
	P1.7–2,9	F1-5	ST-9354	cc41/44	2
	P1.22–1,14	NA^†^	ST-472	cc35	2
	P1.18–1,30–8	F3-3	ST-7460	cc32	1
	P1.7–1,1	F3-3	ST-7460	cc32	1
	P1.22,14	F5-5	ST-213	cc213	1
	P1.22,14	NA^†^	ST-213	cc213	1
	P1.7–2,4–11	F5-9	ST-162	cc162	1
	P1.21,16–36	F5-8	ST-12464	UNK*	1
	P1.21,16–59	F5-8	ST-12464	UNK*	1
	P1.19,13–15	F5-1	ST-336	UNK*	1
	P1.22,9	F5-9	ST-3934	UNK*	1
	P1.17–6,23	F3-36	ST-12465	UNK*	1
	P1.21,16–36	F5-8	ST-13217	UNK*	1
**MenY (n = 7)**	P1.5–2,10–2	F2-13	ST-23	cc23	3
	P1.5–2,10–2	F4-1	ST-23	cc23	1
	P1.5–2,10–2	F4-5	ST-23	cc23	1
	P1.5–1,10–1	F4-1	ST-1655	cc23	1
	P1.5–1,10–1	F3-4	ST-3980	cc167	1
**MenE (n = 1)**	P1.21,26	F5-2	ST-12466	cc60	1
**MenZ (n = 1)**	P1.18–1,3	F5-7	ST-12468	UNK*	1
**NG**^**§**^ **(n = 9)**	P1.22–11,15–25	F5-1	ST-175	cc175	2
	P1.5–2,10–2	F2-13	ST-23	cc23	1
	P1.5–2,10–1	F2-13	ST-23	cc23	1
	P1.22–1,14	F4-1	ST-35	cc35	1
	P1.5,2	F1-7	ST-1383	cc60	1
	P1.5–1,10–4	NA^†^	ST-12469	cc167	1
	P1.19,15	F1-84	ST-414	cc41/44	1
	P1.22–36,25–89	F5-7	ST-12472	UNK*	1

§ Non-groupable.

† Not applicable.

‡ Deleted gene.

* Unknown.

Forty PorA (P1.VR1,VR2) and 23 FetA VR types were identified (Table 1). Moreover, 8.2% (7/85) of isolates showed *fetA* gene deletion (Δ*fetA*). Fifteen PorA and 15 FetA types were found in more than one isolate. The most common finetype combination (P1.VR1,VR2:FVR) was P1.18–4,25:F4-49 (9.4%; 8/85), exclusively found among *cnl*. Three of the 7 (42.9%) MenY showed the P1.5–2,10–2:F2-13 finetype.

As reported in Table 1, 33 Sequence Types (STs), belonging to at least 13 previously known clonal complexes (cc_s_), were determined. The three most common, cc1136 (22.3%; 19/85), cc198 (15.3%; 13/85) and cc53 (14.1%; 12/85), were exclusively found in *cnl* meningococci. MenB isolates were mainly grouped into the cc865 (22.7%; 5/22) and cc41/44 (18.2%; 4/22); MenY into the cc23 (85.7%; 6/7).

One PorA VR1 (P1.18–45), 2 PorA VRs2 (P1.25–88 and P1.25–89) and 2 STs (ST-13193 and ST-13194) have not been previously assigned.

Overall, 64 genotypic profiles were obtained and 53 of them were represented by one isolate; the most frequent profile was *cnl*:P1.18–4,25:F4-49:ST-1136 (cc1136) (8.2%; 7/85).

**Fig 1:**
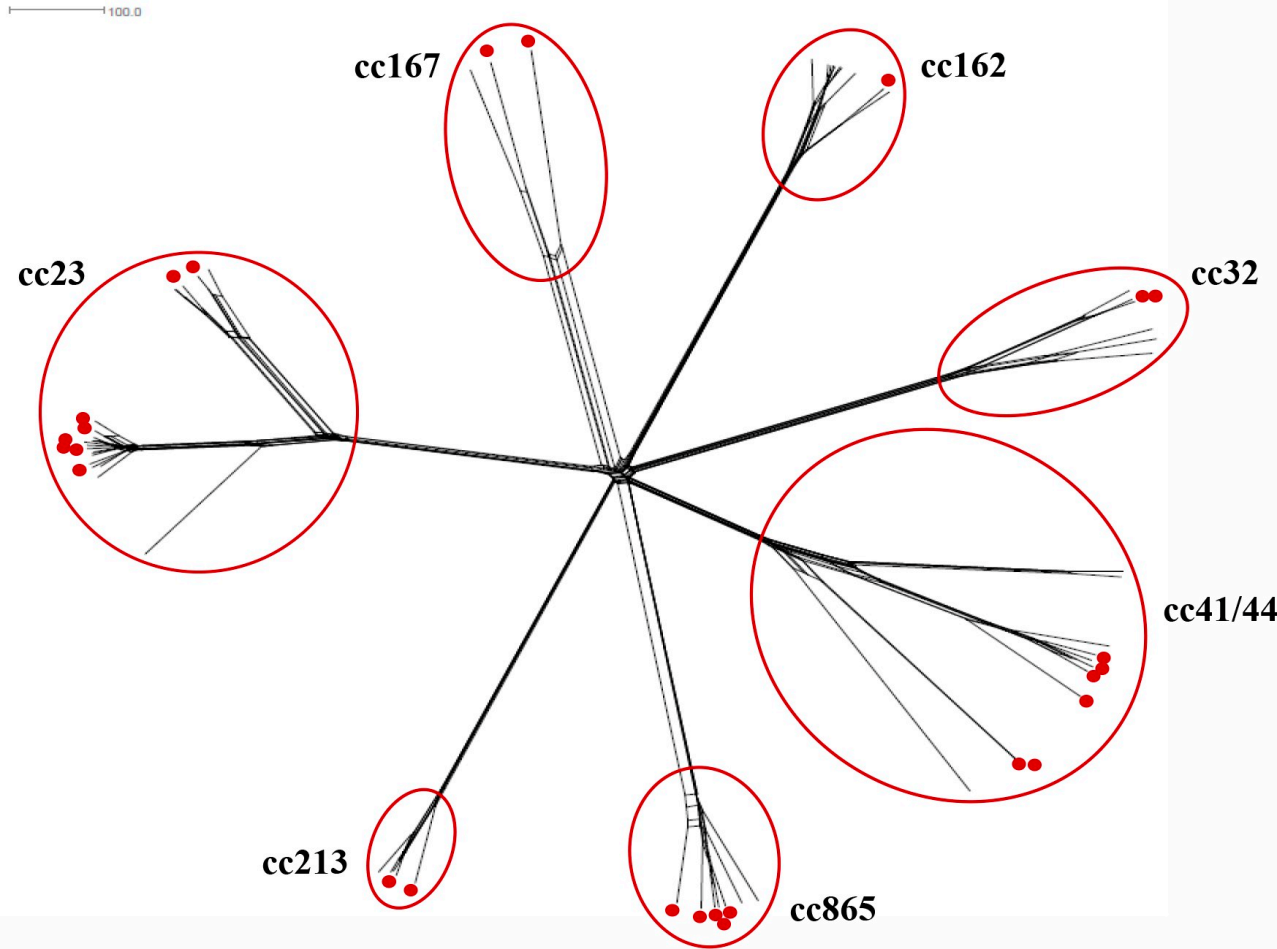
cgMLST analysis of 26 Tuscany carriage isolates and 37 invasive meningococcal isolates collected in Italy.

A comparison between Tuscany’s carriage (*n* = 26) and invasive isolates collected in Italy in the same time period (*n* = 37), belonging to cc23, cc32, cc41/44, cc162, cc213 and cc865 (Table A in S1 Appendix), was performed by cgMLST. Meningococci belonging to the same clonal complex grouped together (Fig 1), and a high similarity between carriage and invasive isolates was found in the main cc23 subcluster (average distance among components of 77 loci).

**Fig 2:**
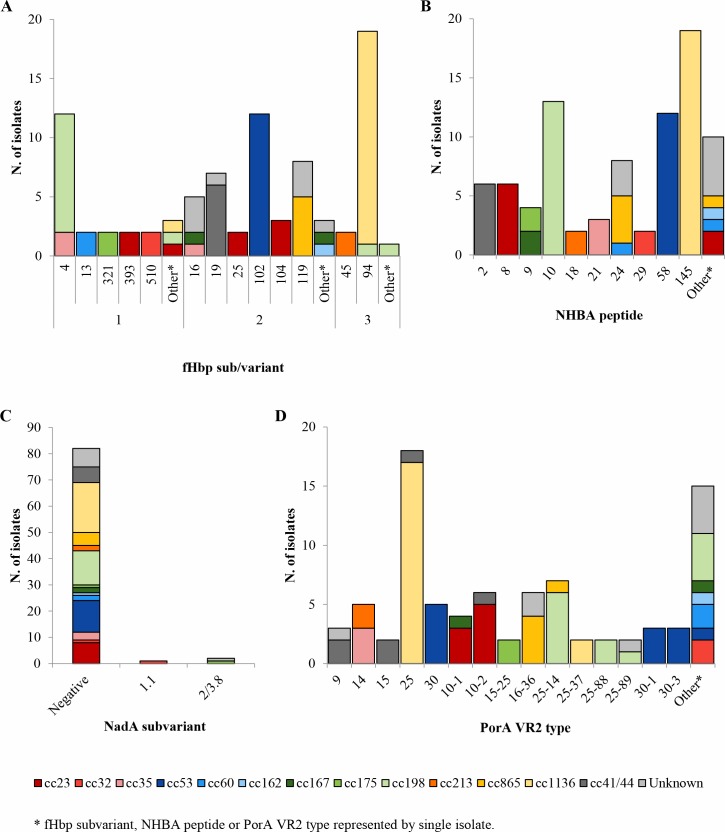
Distribution of 4CMenB vaccine antigens by clonal complex among 85 meningococcal carriage isolates. (A) fHbp variants and subvariants; (B) NHBA peptides; (C) NadA subvariants; (D) PorA VR2 types.

Isolates were further analyzed for the MenB vaccine antigens and for the Bexsero antigen sequence types (Fig 2 and Table B in S1 Appendix).

A complete coding sequence for fHbp was detected for all of them. Variant family 2 was the most common (47.1%; 40/85), followed by variant family 1 (27.1%; 23/85) and variant family 3 (25.9%; 22/85). Twenty peptide subvariants were identified, of which 7 were represented by single isolates. The most frequent subvariant was fHbp-3.94 (22.3%; 19/85) mostly found among cc1136 isolates (94.7%; 18/19) (Fig 2A). None of the analyzed isolates was able to encode the subvariant fHbp-1.1, included in the 4CMenB (Bexsero) formulation. Two MenB:cc213 carriage isolates (2.35%; 2/85) showed the subvariant fHbp-3.45 (A05) (Table B in S1 Appendix), comprised in the bivalent MenB vaccine (Trumenba) formulation.

All the 85 isolates harbored *nhba* gene and 20 NHBA peptides were identified, of which 10 only once. The most frequent peptide was NHBA-145 (22.3%; 19/85), which was exclusively observed in cc1136 isolates (Fig 2B). Six of 85 (7.1%) isolates had genes predicted to encode for the NHBA-2 peptide, contained in the 4CMenB vaccine. All of these belonged to cc41/44, including 4 MenB isolates, 1 *cnl* and 1 NG (Table B in S1 Appendix).

An entire coding sequence for NadA was found in 3 isolates: MenB:cc32, encoding the subvariant NadA-1.1; MenZ:UNK and NG:cc175, encoding the subvariant NadA-2/3.8 (Fig 2C and Table B in S1 Appendix), included in the 4CMenB formulation. Two MenB:cc213 isolates (2.3%; 2/85) were negative for NadA subvariant due to the presence of a frameshift mutation in the *nadA* sequence that would result in phase-off gene expression, while the remaining isolates (94.1%; 80/85) were negative due to a gene deletion or gene disruption by the insertion sequence IS*1301*.

Thirty PorA VR2 types were identified, half of which were present only once. The most common VR2 type was P1.25 (21.2%; 18/85), mainly found in isolates belonging to cc1136 (94.4%; 17/18), (Fig 2D). The PorA VR2 P1.4, component of the 4CMenB vaccine, was not detected.

Of the 47 BASTs identified (Table B in S1 Appendix), only 14 (29.79%) were found in more than one isolate. The most represented was the BAST-657 (18.82%; 16/85), detected in the 35.56% of *cnl* meningococci (16/45).

**Fig 3:**
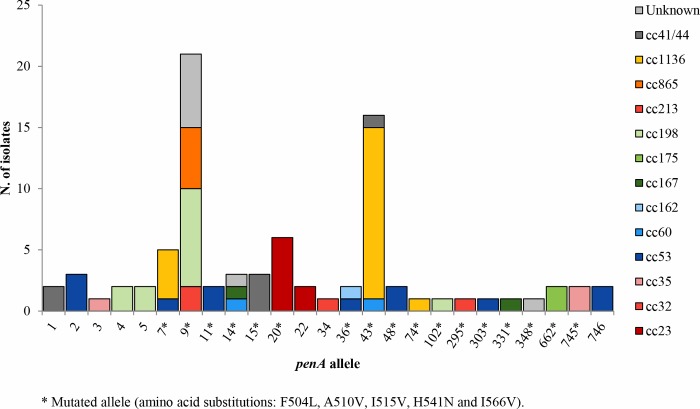
Distribution of *penA* alleles by clonal complex among 85 meningococcal carriage isolates.

Twenty-five *penA* alleles were defined; *penA9* was the most frequent (24.7%; 21/85) and found mainly among cc198 isolates (38.1%; 8/21) (Fig 3). More than 82% (70/85) of the isolates showed mutated *penA* alleles (7, 9, 11, 14, 15, 20, 36, 43, 48, 74, 102, 295, 303, 331, 348, 662 and 745) coding for all the following five amino acid substitutions: F504L, A510V, I515V, H541N and I566V.

Finally, the molecular characterization of the denitrification pathway genes, *aniA* and *norB*, revealed that both coding sequences were complete in 71.8% (61/85) of the isolates, including 100% of the *cnl* isolates (45/45), 45.4% of the MenB (10/22), 55.6% of the NG (5/9) and the single MenZ (Table 2).

**Table 2:** Distribution of *aniA* and *norB* coding sequences by capsular group among 85 meningococcal carriage isolates.

	*cnl*	MenB	MenE	MenY	MenZ	NG^§^	N. of isolates
**Intact *aniA* and *norB***	45	10	0	0	1	5	61
**Intact *aniA*—disrupted/deleted *norB***	0	0	0	0	0	0	0
**Intact *norB*—disrupted/deleted *aniA***	0	11	1	7	0	4	23
**Disrupted/deleted *aniA* and *norB***	0	1	0	0	0	0	1

§ Non-groupable.

## Discussion

Since 2012, a significant increase in the proportion of MenC cases has been observed in Italy, making it one of the most frequent serogroups causing IMD in the country [23].

Due to a MenC:cc11 outbreak occurred in Tuscany Region [15–17] a cross-sectional carriage survey was conducted in order to evaluate how the *N*. *meningitidis* carriage played a role in the strain spread during the outbreak [18, 19].

Here, the genomic analysis was carried out on cultivated carriage isolates to analyze their main molecular traits. Unfortunately, the unique 4 MenC:cc11 carriers, previously described by Miglietta et al. [19], resulted positive only by molecular methods and the isolates were not available for WGS analysis.

Overall, the majority of carriage isolates subjected to WGS were *cnl* (53%). Moreover, in agreement with previous carriage studies [24, 25], MenB was the most frequently identified serogroup among groupable isolates (71%), followed by MenY (23%). Of note, 2 MenB and 2 MenY identified by Miglietta et al. [19], resulted non-groupable by WGS due to the lack of *cps* biosynthetic genes.

Despite the high heterogeneity, the carriage isolates clustered by capsular serogroup and clonal complex. The three prevalent cc_s_, the cc1136, the cc198 and the cc53, were already associated with *cnl* meningococcal carriage [26]. Interestingly, strains belonging to cc41/44, cc32 and cc23, three of the main cc_s_ associated with IMD in Italy and in Europe [27–29], were also identified among the carriage isolates collected during the Tuscan epidemic.

The cgMLST analysis grouped the isolates regardless of invasiveness, and highlighted close similarities among cc23 carriage and invasive meningococci, as expected [30]. Y:P1.5–2,10–2:F2-13:ST-23 (cc23) (*n* = 3) and Y:P1.5–1,10–1:F4-1:ST-1655 (cc23) (*n* = 1), here identified, were associated to a high proportion of MenY IMD cases in Italy and England and Wales, respectively [31, 32]. During the last years, many European countries reported an increase in the incidence of MenY IMD [33, 34]. This may be in part explained by the capability of MenY to successfully colonize the pharynx [30, 35] and then favoring the spread in the population.

A high variability with respect to the MenB vaccine antigen-encoding genes was also found, as suggested by the BASTs results. Only eight carriage isolates presented at least one 4CMenB (Bexsero) matching gene and two isolates showed the gene encoding the A subvariant included in the bivalent MenB vaccine (Trumenba). Due to the lack of MATS-ELISA assay [36], the results do not permit to estimate the vaccine coverage among the examined meningococci. However, recently, a new genetic Meningococcal Antigen Typing System (gMATS) was set up to correlate antigen genotypes and coverage estimates by MATS [37]. Some gMATS predictors of coverage identified by Muzzi et al. [37] were also found in this work: fHbp peptides 4 and 510; NHBA peptides 2, 10, 20 and 21. Consequently, 29% of the carriage isolates were estimated to be covered by 4CMenB (Bexsero) vaccine.

Of note, more than 82% of the Tuscany’s carriage isolates harbored mutated *penA* alleles encoding a modified penicillin binding protein 2 that is involved in reducing susceptibility to penicillin G [38]. In the last two decades, this phenotype has been increasingly reported in several countries, including Italy, where it represents the majority of meningococci causing invasive disease [14, 38, 39].

The ability of meningococci to adapt to oxygen deficiency, even though not essential for meningococcal survival in the pharynx [40], may facilitate new niche of adaptation [41] through the expression of the *aniA* and *norB* genes [10, 40, 41]. More than 71% of the carriage isolates showed an intact coding sequence for both of the genes, suggesting that the transmission chain may include low-oxic environments, as the urethra [41]. However, the results need to be further investigated in order to define a precise role of the denitrification pathway in the survival of carriage isolates associated to specific genomic characteristics.

To conclude, this is the first genomic analysis of meningococcal carriage isolates collected during an outbreak in Italy. To this regard, it should be underlined that WGS offers a greater degree of accuracy to define the main genomic traits of the strains, including capsular group determination, compared to phenotypic and/or polymerase chain reaction assay, which frequently misclassify carriage isolates [42, 43].

Overall, this study provided evidence of an extensive diversity among meningococcal carriage isolates during the MenC:cc11 outbreak in Tuscany. Even though an outbreak is a multifactorial event resulting from changes in host-pathogen interactions, the results suggest a quite low recovery degree of the MenC:cc11 in the pharynx, as already described in previous carriage surveys [19, 44], and are consistent with a high transmission rate of MenC:cc11strain [45].

## Supporting information

S1 Appendix This appendix contains Tables A and B.
(DOC)
