# Supplementary material for: Genomic analysis of Neisseria meningitidis carriage isolates during an outbreak of serogroup C clonal complex 11, Tuscany, Italy
Source: PLoS One. 2019 May 28;14(5):e0217500. doi: 10.1371/journal.pone.0217500 (PMC6538176; doi:10.1371/journal.pone.0217500)
Supplement: S1 Appendix — This appendix contains Tables A and B. (DOC) [file pone.0217500.s001.doc]

**Table A.** Identification codes in pubMLST.org database and genotypic profiles of the 37 meningococcal invasive isolates included in the cgMLST comparison.

§ Not applicable

| **ID number** | **Serogroup** | **PorA type** | **FetA type** | **Sequence Type** | **clonal complex** |
| --- | --- | --- | --- | --- | --- |
| **http://pubmlst.org/Neisseria** |  |  |  | **(ST)** | **(cc)** |
| 41655 | B | P1.7-2,4 | F1-2 | ST-1403 | cc41/44 |
| 41656 | Y | P1.5-2,10-58 | NA^§^ | ST-23 | cc23 |
| 41658 | B | P1.5-2,10 | F5-1 | ST-34 | cc32 |
| 44975 | Y | P1.5-2,10-2 | F2-13 | ST-23 | cc23 |
| 52930 | Y | P1.5-2,10-2 | F2-13 | ST-23 | cc23 |
| 52931 | B | P1.5-1,10-4 | F5-36 | ST-1624 | cc167 |
| 52935 | Y | P1.5-2,10-2 | F2-13 | ST-23 | cc23 |
| 52936 | Y | P1.5-1,2-2 | F4-1 | ST-3587 | cc23 |
| 52937 | B | P1.7,16-36 | F5-8 | ST-3327 | cc865 |
| 52938 | B | P1.22,14 | F3-6 | ST-162 | cc162 |
| 52940 | B | P1.22,14 | F3-6 | ST-162 | cc162 |
| 52943 | B | P1.22,14 | F5-9 | ST-162 | cc162 |
| 52945 | B | P1.5,10-2 | F1-84 | ST-414 | cc41/44 |
| 52946 | B | P1.22,14 | F5-5 | ST-213 | cc213 |
| 52948 | B | P1.7-2,4 | F5-9 | ST-9293 | cc162 |
| 52949 | B | P1.22,14 | F3-6 | ST-162 | cc162 |
| 52950 | B | P1.19,15 | F5-1 | ST-8758 | cc32 |
| 52951 | C | P1.21,16-36 | F5-5 | ST-NA^§^ | cc865 |
| 52953 | B | P1.22,14-6 | F1-5 | ST-6349 | cc41/44 |
| 52954 | B | P1.18,25 | F1-84 | ST-414 | cc41/44 |
| 52956 | Y | P1.5-2,10-2 | F2-13 | ST-23 | cc23 |
| 52957 | B | P1.19,15 | F1-24 | ST-34 | cc32 |
| 52958 | B | P1.22,14 | F3-6 | ST-162 | cc162 |
| 52960 | B | P1.18-1,30-8 | F3-3 | ST-13428 | cc32 |
| 84056 | Y | P1.5-2,10-2 | F2-13 | ST-9253 | cc23 |
| 84057 | Y | P1.5-2,10-2 | F4-12 | ST-23 | cc23 |
| 84060 | B | P1.22,14 | F3-6 | ST-162 | cc162 |
| 84061 | B | P1.22,14 | F3-6 | ST-10812 | cc162 |
| 84062 | B | P1.7-2,4 | F1-5 | ST-1403 | cc41/44 |
| 84063 | B | P1.22,14 | F3-6 | ST-162 | cc162 |
| 84065 | Y | P1.5-1,10-1 | F4-1 | ST-1655 | cc23 |
| 84066 | Y | P1.5-2,10-40 | F4-1 | ST-23 | cc23 |
| 84068 | Y | P1.5-2,10-2 | F4-1 | ST-23 | cc23 |
| 84069 | B | P1.22,14 | F5-5 | ST-213 | cc213 |
| 84070 | B | P1.22,14 | F5-5 | ST-213 | cc213 |
| 84072 | Y | P1.5-2,10-1 | F4-1 | ST-23 | cc23 |
| 84073 | Y | P1.5-2,10-2 | F5-13 | ST-23 | cc23 |

**Table B.** Identification codes in pubMLST.org database, capsular groups, multilocus sequence types, 4CMenB (Bexsero®) protein variants and Bexsero® antigen sequence types (BASTs) of 85 meningococcal carriage isolates.

| **ID number**  **http://pubmlst.org/Neisseria** | **Genogroup** | **Sequence Type (ST)** | **clonal complex (cc)** | **fHbp**  **Novartis variant** | **NHBA peptide** | ***nadA* presence** | **NadA variant** | **NadA peptide** | **PorA type** | **BAST** |
| --- | --- | --- | --- | --- | --- | --- | --- | --- | --- | --- |
| 47062 | *cnl* | ST-1136 | cc1136 | 3.94 | 145 | - | - | - | P1.18-4,25 | 657 |
| 47064 | B | ST-9354 | cc41/44 | 2.19 | 2 | - | - | - | P1.7-2,9 | 3097 |
| 47065 | *cnl* | ST-1136 | cc1136 | 3.94 | 145 | - | - | - | P1.18-4,25 | 657 |
| 47066 | *cnl* | ST-823 | cc198 | 1.4 | 10 | - | - | - | P1.18,25-54 | 3098 |
| 47067 | *cnl* | ST-823 | cc198 | 1.4 | 10 | - | - | - | P1.18,25-32 | 2031 |
| 47068 | B | ST-213 | cc213 | 3.45 | 18 | + | 4/5 | FS^†^ | P1.22,14 | 224 |
| 47069 | *cnl* | ST-198 | cc198 | 3.94 | 10 | - | - | - | P1.18,25-1 | 661 |
| 47071 | *cnl* | ST-823 | cc198 | 1.4 | 10 | - | - | - | P1.18,25-14 | 597 |
| 47072 | B | ST-12464 | UNK^‡^ | 2.119 | 24 | - | - | - | P1.21,16-36 | 1755 |
| 47073 | *cnl* | ST-53 | cc53 | 2.102 | 58 | - | - | - | P1.7,30-3 | 635 |
| 47074 | *cnl* | ST-1136 | cc1136 | 3.94 | 145 | - | - | - | P1.18-4,25 | 657 |
| 47075 | *cnl* | ST-13193 | cc1136 | 3.94 | 145 | - | - | - | P1.18-4,25-37 | 2511 |
| 47076 | *cnl* | ST-1136 | cc1136 | 3.94 | 145 | - | - | - | P1.18-4,25 | 657 |
| 47078 | B | ST-414 | cc41/44 | 2.19 | 2 | - | - | - | P1.19,15 | 644 |
| 47079 | *cnl* | ST-823 | cc198 | 1.4 | 10 | - | - | - | P1.18,25-14 | 597 |
| 47082 | *cnl* | ST-1136 | cc1136 | 3.94 | 145 | - | - | - | P1.18-4,25 | 657 |
| 47083 | *cnl* | ST-53 | cc53 | 2.102 | 58 | - | - | - | P1.7,30 | 637 |
| 47084 | *cnl* | ST-823 | cc198 | 1.4 | 10 | - | - | - | P1.18,25-14 | 597 |
| 47085 | *cnl* | ST-198 | cc198 | 3.319 | 10 | - | - | - | P1.18-45,25-11 | 3099 |
| 47086 | B | ST-12465 | UNK^‡^ | 2.16 | 669 | - | - | - | P1.17-6,23 | 811 |
| 47087 | *cnl* | ST-11167 | cc53 | 2.102 | 58 | - | - | - | P1.7-2,30-1 | 854 |
| 47088 | *cnl* | ST-823 | cc198 | 1.4 | 10 | - | - | - | P1.18,25-88 | 3100 |
| 47089 | *cnl* | ST-1136 | cc1136 | 3.94 | 145 | - | - | - | P1.18-4,25 | 657 |
| 47090 | E | ST-12466 | cc60 | 1.13 | 664 | - | - | - | P1.21,26 | NA^*^ |
| 47091 | B | ST-7460 | cc32 | 1.510 | 29 | - | - | - | P1.18-1,30-8 | 3048 |
| 47092 | *cnl* | ST-53 | cc53 | 2.102 | 58 | - | - | - | P1.7-2,30-2 | 1266 |
| 47093 | *cnl* | ST-1136 | cc1136 | 3.94 | 145 | - | - | - | P1.18-4,25 | 657 |
| 47094 | B | ST-12464 | UNK^‡^ | 2.119 | 24 | - | - | - | P1.21,16-59 | 3101 |
| 47096 | *cnl* | ST-823 | cc198 | 11.102 | 10 | - | - | - | P1.18,25-14 | 3102 |
| 47098 | Z | ST-12468 | UNK^‡^ | 2.16 | 101 | + | 2/3 | 8 | P1.18-1,3 | 2038 |
| 47099 | *cnl* | ST-53 | cc53 | 2.102 | 58 | - | - | - | P1.7,30 | 637 |
| 47100 | *cnl* | ST-1136 | cc1136 | 3.94 | 145 | - | - | - | P1.18-4,25-37 | 2511 |
| 47101 | NG^§^ | ST-175 | cc175 | 1.321 | 9 | + | 2/3 | 8 | P1.22-11,15-25 | 3103 |
| 47102 | B | ST-472 | cc35 | 1.4 | 21 | - | - | - | P1.22-1,14 | 2999 |
| 47103 | *cnl* | ST-823 | cc198 | 1.4 | 10 | - | - | - | P1.18,25-14 | 597 |
| 47104 | NG^§^ | ST-35 | cc35 | 2.16 | 21 | - | - | - | P1.22-1,14 | 257 |
| 47105 | *cnl* | ST-1136 | cc1136 | 3.94 | 145 | - | - | - | P1.18-4,25 | 657 |
| 47106 | B | ST-3934 | UNK^‡^ | 2.19 | 17 | - | - | - | P1.22,9 | 267 |
| 47107 | *cnl* | ST-1136 | cc1136 | 3.94 | 145 | - | - | - | P1.18-4,25 | 657 |
| 47108 | B | ST-7460 | cc32 | 1.510 | 29 | + | 1 | 1 | P1.7-1,1 | 151 |
| 47109 | Y | ST-23 | cc23 | 1.437 | 8 | - | - | - | P1.5-2,10-2 | 3105 |
| 47111 | Y | ST-23 | cc23 | 2.25 | 1272 | - | - | - | P1.5-2,10-2 | 3112 |
| 47113 | *cnl* | ST-1136 | cc1136 | 3.94 | 145 | - | - | - | P1.18-4,25 | 657 |
| 47114 | Y | ST-3980 | cc167 | 2.95 | 9 | - | - | - | P1.5-1,10-1 | 3113 |
| 47115 | NG^§^ | ST-175 | cc175 | 1.321 | 9 | - | - | - | P1.22-11,15-25 | 3133 |
| 47116 | B | ST-336 | UNK^‡^ | 2.106 | 122 | - | - | - | P1.19,13-15 | 3160 |
| 47117 | NG^§^ | ST-414 | cc41/44 | 2.19 | 2 | - | - | - | P1.19,15 | 644 |
| 47118 | B | ST-3327 | cc865 | 2.119 | 24 | - | - | - | P1.21,16-36 | 1755 |
| 47119 | Y | ST-23 | cc23 | 2.104 | 8 | - | - | - | P1.5-2,10-2 | 275 |
| 47120 | NG^§^ | ST-23 | cc23 | 1.393 | 8 | - | - | - | P1.5-2,10-2 | 3043 |
| 47121 | NG^§^ | ST-12469 | cc167 | 2.16 | 9 | - | - | - | P1.5-1,10-4 | 863 |
| 47122 | B | ST-213 | cc213 | 3.45 | 18 | + | 4/5 | FS^†^ | P1.22,14 | 224 |
| 47123 | Y | ST-1655 | cc23 | 2.25 | 7 | - | - | - | P1.5-1,10-1 | 221 |
| 47124 | Y | ST-23 | cc23 | 2.104 | 8 | - | - | - | P1.5-2,10-2 | 275 |
| 47126 | B | ST-162 | cc162 | 2.21 | 20 | - | - | - | P1.7-2,4-11 | 3161 |
| 47127 | *cnl* | ST-1136 | cc1136 | 3.94 | 145 | - | - | - | P1.18-4,25 | 657 |
| 47128 | B | ST-3327 | cc865 | 2.119 | 24 | - | - | - | P1.18,25-14 | 1755 |
| 47129 | *cnl* | ST-1136 | cc1136 | 3.94 | 145 | - | - | - | P1.18-4,25 | 657 |
| 47130 | *cnl* | ST-1136 | cc1136 | 3.94 | 145 | - | - | - | P1.18-4,25 | 657 |
| 47132 | *cnl* | ST-12470 | cc1136 | 3.94 | 145 | - | - | - | P1.18-4,25 | 657 |
| 47134 | *cnl* | ST-13194 | cc53 | 2.102 | 58 | - | - | - | P1.7-2,30-3 | 1745 |
| 47135 | Y | ST-23 | cc23 | 1.393 | 8 | - | - | - | P1.5-2,10-2 | 3043 |
| 47136 | *cnl* | ST-11738 | cc1136 | 11.077 | 145 | - | - | - | P1.18-4,25 | 3162 |
| 47137 | *cnl* | ST-53 | cc53 | 2.102 | 58 | - | - | - | P1.7-2,30 | 1744 |
| 47138 | *cnl* | ST-53 | cc53 | 2.102 | 58 | - | - | - | P1.7-2,30-3 | 1745 |
| 47139 | B | ST-414 | cc41/44 | 2.19 | 2 | - | - | - | P1.18,25 | 1986 |
| 47141 | *cnl* | ST-823 | cc198 | 1.4 | 10 | - | - | - | P1.18,25-89 | 3163 |
| 47142 | *cnl* | ST-1136 | cc1136 | 3.94 | 145 | - | - | - | P1.18-4,25 | 657 |
| 47143 | *cnl* | ST-1136 | cc1136 | 3.94 | 145 | - | - | - | P1.18-4,25 | 657 |
| 47144 | B | ST-3327 | cc865 | 2.119 | 1273 | - | - | - | P1.21,16-36 | 3164 |
| 47145 | *cnl* | ST-823 | cc198 | 1.4 | 10 | - | - | - | P1.18,25-88 | 3100 |
| 47146 | *cnl* | ST-53 | cc53 | 2.102 | 58 | - | - | - | P1.7,30 | 637 |
| 47148 | *cnl* | ST-12471 | cc41/44 | 2.19 | 2 | - | - | - | P1.18,10-2 | 3166 |
| 47150 | B | ST-13217 | UNK^‡^ | 2.119 | 24 | - | - | - | P1.21,16-36 | 1755 |
| 47151 | *cnl* | ST-53 | cc53 | 2.102 | 58 | - | - | - | P1.7,30-1 | 1978 |
| 47152 | *cnl* | ST-823 | cc198 | 1.4 | 10 | - | - | - | P1.18,25-14 | 597 |
| 47153 | NG^§^ | ST-23 | cc23 | 2.104 | 8 | - | - | - | P1.5-2,10-1 | 3167 |
| 47154 | *cnl* | ST-11167 | cc53 | 2.102 | 58 | - | - | - | P1.7-2,30-1 | 854 |
| 47155 | B | ST-3327 | cc865 | 2.119 | 24 | - | - | - | P1.5-2,16-36 | 1755 |
| 47156 | NG^§^ | ST-12472 | UNK^‡^ | 2.16 | 601 | - | - | - | P1.22-36,25-89 | 3168 |
| 47158 | B | ST-472 | cc35 | 1.4 | 21 | - | - | - | P1.22-1,14 | 2999 |
| 47159 | B | ST-3327 | cc865 | 2.119 | 24 | - | - | - | P1.21,16-36 | 1755 |
| 47160 | B | ST-9354 | cc41/44 | 2.19 | 2 | - | - | - | P1.7-2,9 | 3097 |
| 47162 | *cnl* | ST-12473 | cc53 | 2.102 | 58 | - | - | - | P1.7,30 | 637 |
| 47164 | NG^§^ | ST-1383 | cc60 | 1.13 | 24 | - | - | - | P1.5,2 | 237 |

§ Non-groupable.

†Frameshift mutation in the coding region resulting in a premature stop codon.

‡Unknown

*Not applicable.
